# Supplementary material for: Multiciliated cells use filopodia to probe tissue mechanics during epithelial integration in vivo
Source: Nat Commun. 2022 Oct 28;13:6423. doi: 10.1038/s41467-022-34165-0 (PMC9616887; doi:10.1038/s41467-022-34165-0)
Supplement: Supplementary file 1 — Supplementary Information [file 41467_2022_34165_MOESM1_ESM.pdf]

Supplementary Information

Multiciliated cells use filopodia to probe tissue mechanics during epithelial integration *in vivo*

Guilherme Ventura, Aboutaleb Amiri, Raghavan Thiagarajan, Mari Tolonen, Amin Doostmohammadi, Jakub Sedzinski

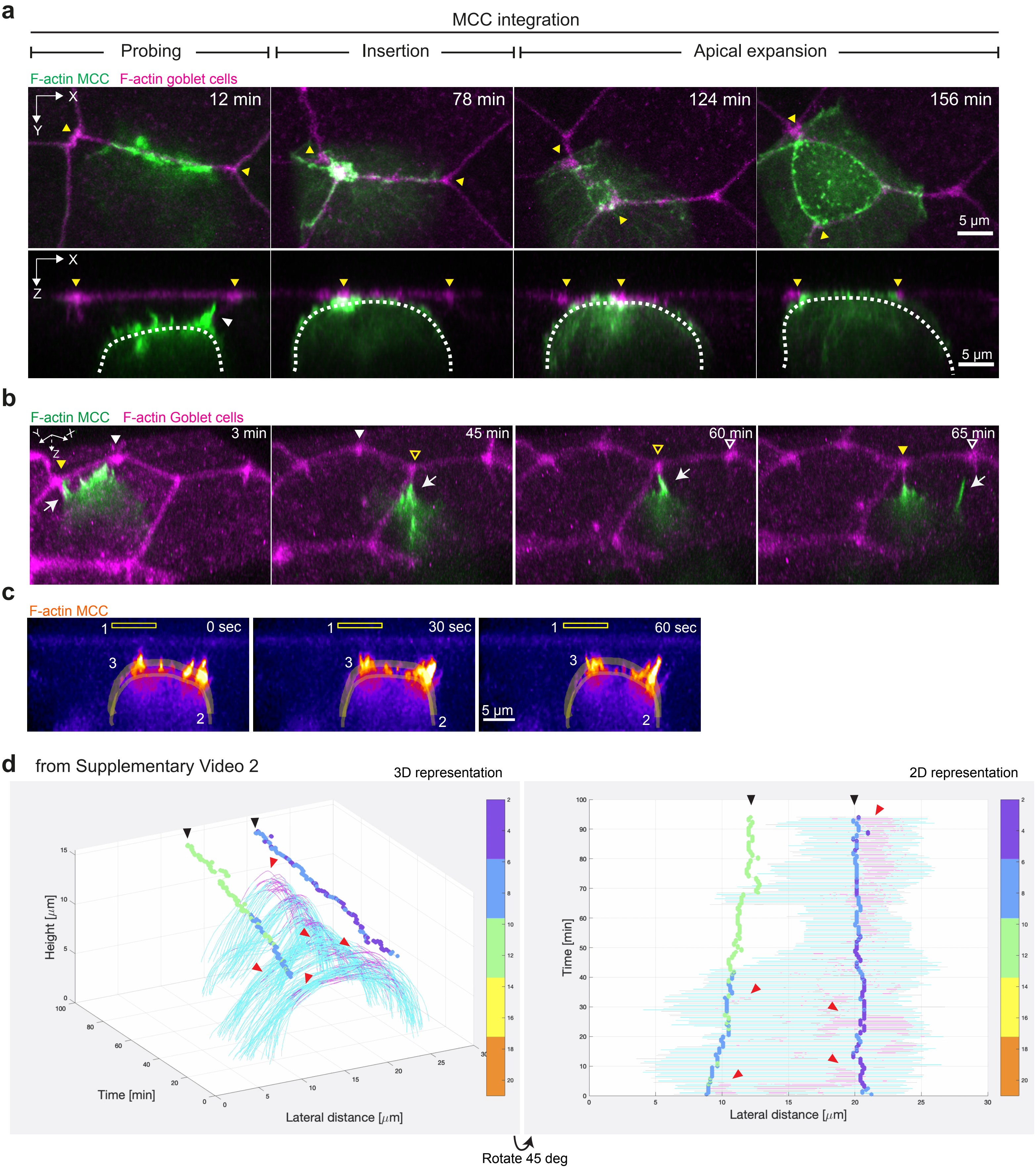
a
MCC integration
Probing
Insertion
Apical expansion
F-actin MCC
F-actin goblet cells
12 min
78 min
124 min
156 min
5 µm
b
F-actin MCC
F-actin Goblet cells
3 min
45 min
60 min
65 min
c
F-actin MCC
0 sec
30 sec
60 sec
5 µm
d
from Supplementary Video 2
3D representation
2D representation
Height [µm]
Time [min]
Lateral distance [µm]
Rotate 45 deg

Supplementary Fig. 1 Filopodia dynamics during the probing phase of MCC integration. Integrating MCCs express F-actin marker (LifeAct, pseudo-colored in green) under MCC-specific α-tubulin promoter while overlying goblet cells express F-actin marker (Utrophin-RFP, pseudo-colored in magenta) under goblet cell-specific nectin promoter. **a,** Snapshots of the different steps of the MCC integration process: probing (when MCCs move into the superficial layer), insertion (when MCCs are stably positioned at an epithelial vertex) and apical expansion (when MCCs join the tissue by expanding their apical domain). Yellow arrowheads mark epithelial vertices. In the corresponding orthogonal (XZ) projections, yellow arrowheads mark epithelial vertices and the white arrow indicates a F-actin filopodium. White dotted lines outline the cortex of the integrating MCC. Scale bars: 5 µm. **b,** 3D rendering of integrating MCC interacting with different vertices (filopodia marked by arrowheads)(MCC from Fig. 1b-d and Supplementary Video 1). **c,** Representation of filopodia dynamics quantification. Different regions of interest (ROIs) corresponding to the background (1), cortex (2) and filopodia (3) are drawn and used to extract F-actin intensities (See Methods). Scale bar: 5 µm. **d,** Representation of different modes of visualization of filopodia activity (stills adapted from Supplementary Video 2). F-actin filopodia extended by a single MCC are labeled in magenta while the MCC leading-edge is labeled in cyan. The overlying vertices are represented by the vertical tracks marked by black arrowheads, color-coded for the distance between the MCC tip and the left or right vertex during the probing phase. Lateral distance in µm describes the distance migrated in XY, whereas Height in µm describes the distance migrated in the axial direction. Red arrowheads mark filopodia-vertex interactions.

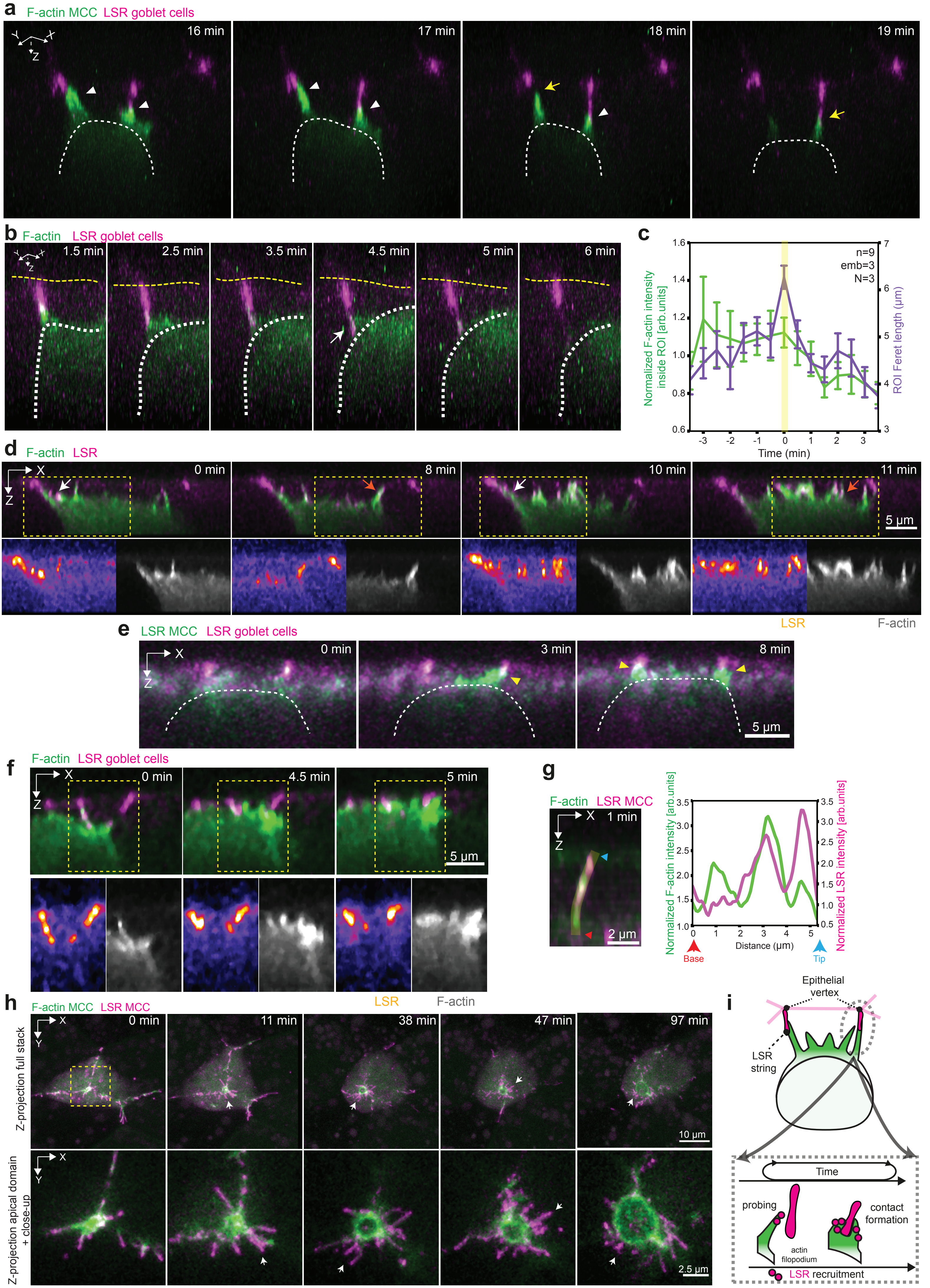
a
F-actin MCC LSR goblet cells
16 min
17 min
18 min
19 min
b
F-actin LSR goblet cells
1.5 min
2.5 min
3.5 min
4.5 min
5 min
6 min
c
n=9
emb=3
N=3
Normalized F-actin intensity inside ROI [arb.units]
ROI Feret length (µm)
Time (min)
d
F-actin LSR
0 min
8 min
10 min
11 min
5 µm
LSR
F-actin
e
LSR MCC LSR goblet cells
0 min
3 min
8 min
5 µm
f
F-actin LSR goblet cells
0 min
4.5 min
5 min
5 µm
LSR
F-actin
g
F-actin LSR MCC
1 min
2 µm
Normalized F-actin intensity [arb.units]
Normalized LSR intensity [arb.units]
Distance (µm)
Base
Tip
h
F-actin MCC LSR MCC
Z-projection full stack
0 min
11 min
38 min
47 min
97 min
10 µm
Z-projection apical domain + close-up
2.5 µm
i
Epithelial vertex
LSR string
Time
probing
actin filopodium
contact formation
LSR recruitment

Supplementary Fig. 2 Filopodia interact directly with epithelial vertices. a, 3D rendering of integrating MCC during the probing phase. The MCC (expressing α-tubulin::LifeAct-RFP, pseudo-colored in green) uses filopodia to attach to the vertices of epithelial goblet cells (expressing nectin::LSR-RFP, pseudo-colored in magenta). White arrowheads depict filopodia-vertex contact and yellow arrows depict contact retraction. **b**, 3D rendering from Fig. 2e of filopodium pulling on vertex (marked by white arrow). White dotted line outlines the cortex of integrating MCC and the yellow dotted line outlines the top of the superficial epithelium. **c**, Epithelial vertex-pulling quantification. Average MCC F-actin intensity (green) and vertex length (purple) during pulling and retraction. T=0 marks the vertex length maxima. Data show mean ± SEM (n=9 pulling events from 3 embryos from N=3 experiments). **d,** Orthogonal (XZ) projections of integrating MCC (expressing α-tubulin::LifeAct-RFP, pseudo-colored in green) expressing LSR-3xGFP (pseudo-colored in magenta). LSR-3xGFP is recruited to the contact points between the MCC and the vertex (white arrows, t=0 min and t= 10 min) and to filopodia (orange arrows, t=8 min and t=11 min). Yellow boxes mark insets with separate channels. Scale bar: 5 µm. **e,** Orthogonal (XZ) projections of integrating MCC expressing α-tubulin::LSR-GFP (pseudo-colored in green) interacting with vertices labeled with LSR-RFP expressed under nectin promoter (pseudo-colored in magenta). Yellow arrowheads mark LSR-LSR co-localization. Scale bar: 5 µm. **f,** Orthogonal (XZ) projections of integrating MCC expressing α-tubulin::LifeAct-RFP interacting with vertices labeled by LSR-GFP (pseudo-colored in magenta) under nectin promoter expression. Insets depict F-actin recruitment in the MCC to stabilize the new contact. Scale bar: 5 µm. **g,** Plot profile of F-actin and LSR along filopodium from Fig. 2d. Normalized fluorescence intensity values for F-actin and LSR are plotted from the base to the tip of filopodium. Scale bar: 2.5 µm. **h,** Image sequence of LSR-overexpressing MCC. The apically expanding MCC expresses α-tubulin::LifeAct-RFP (pseudo-colored in green), and α-tubulin::LSR-RFP (pseudo-colored in magenta). Ectopic protrusions are marked with white arrows. Top row: Full projection. The yellow box marks inset for the bottom row, with a close-up on the apical domain. **i**, Schematics representing LSR recruitment to the leading edge of integrating MCC.

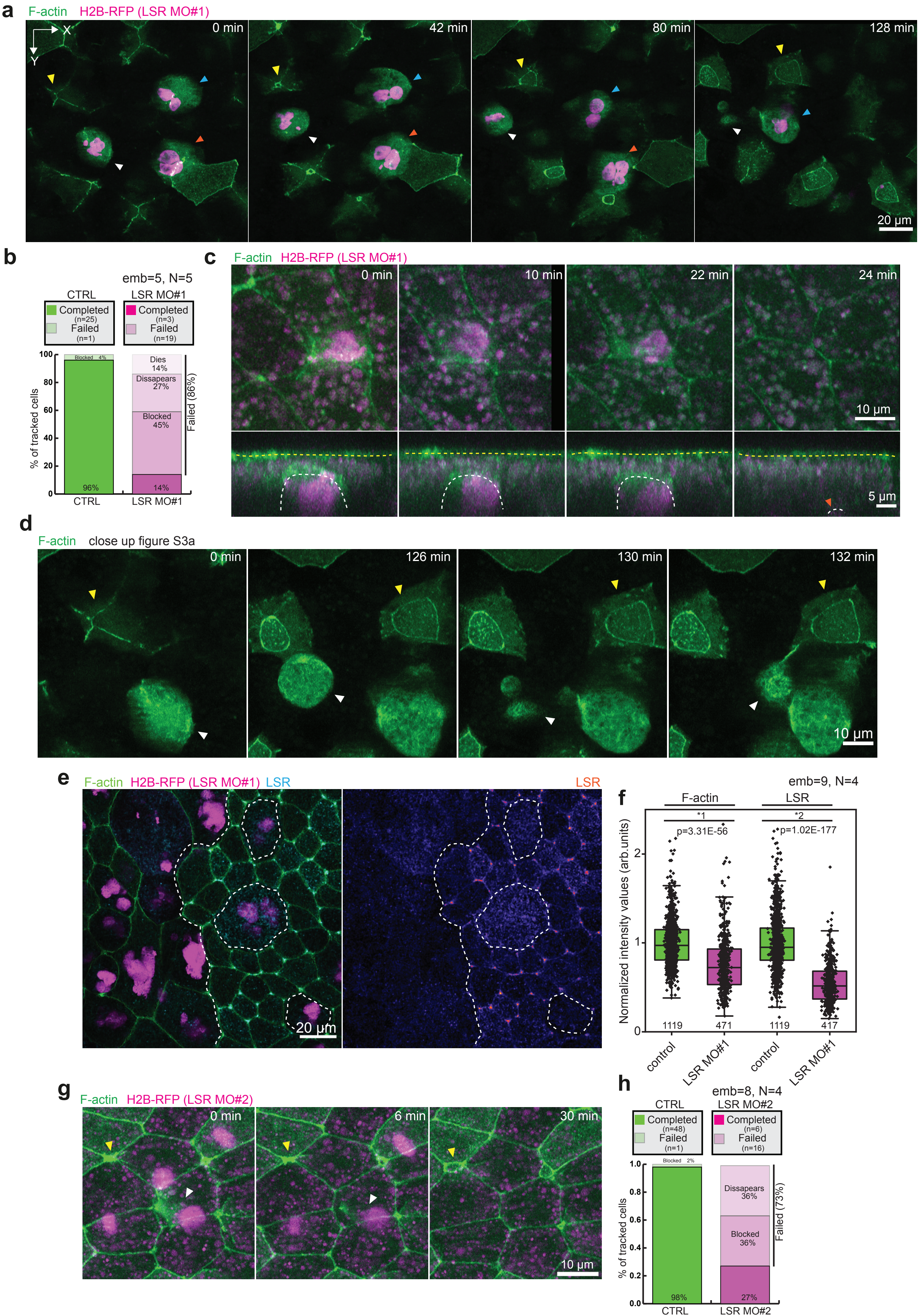

a
F-actin H2B-RFP (LSR MO#1)
X
Y
0 min
42 min
80 min
128 min
20 µm
b
emb=5, N=5
CTRL
Completed (n=25)
Failed (n=1)
LSR MO#1
Completed (n=3)
Failed (n=19)
% of tracked cells
Blocked 4%
96%
Dies 14%
Dissapears 27%
Blocked 45%
14%
Failed (86%)
CTRL
LSR MO#1
c
F-actin H2B-RFP (LSR MO#1)
0 min
10 min
22 min
24 min
10 µm
5 µm
d
F-actin close up figure S3a
0 min
126 min
130 min
132 min
10 µm
e
F-actin H2B-RFP (LSR MO#1) LSR
LSR
20 µm
f
emb=9, N=4
F-actin
LSR
*1
p=3.31E-56
*2
p=1.02E-177
Normalized intensity values (arb.units)
1119
471
1119
417
control
LSR MO#1
control
LSR MO#1
g
F-actin H2B-RFP (LSR MO#2)
0 min
6 min
30 min
10 µm
h
emb=8, N=4
CTRL
Completed (n=48)
Failed (n=1)
LSR MO#2
Completed (n=6)
Failed (n=16)
% of tracked cells
Blocked 2%
98%
Dissapears 36%
Blocked 36%
27%
Failed (73%)
CTRL
LSR MO#2

Supplementary Fig. 3 LSR regulates MCC integration. a,c,d, Control and LSR-depleted cells (LSR MO#1, marked with H2B-RFP, pseudo-colored in magenta) expressing LifeAct-GFP (pseudo-colored in green). **a,** Image sequence of control MCC (yellow arrowhead) and LSR-depleted MCCs. LSR-depleted cells fail to integrate (blocked, cyan arrowhead), migrate back inside the tissue (disappear, orange arrowhead) or undergo cell death (dies, white arrowhead). Scale bar: 20 μm. **b,** Quantification of integration success rates. LSR-depleted MCCs are either blocked, disappear, or die (nWT= 26 cells, nLSRMO#1 = 22 cells from 5 embryos, N=5 experiments). **c,** Image sequence depicting disappearance of LSR-depleted MCC (marked by orange arrowhead). **d,** Image sequence (close-up of figure S3a) depicting cell death of LSR-depleted MCC (marked by white arrowhead). A control MCC is marked by a yellow arrowhead. Scale bar: 10 μm. **e,** Immunofluorescence images of LSR-stained superficial epithelial layer (F-actin pseudo-colored in green and LSR pseudo-colored in cyan). The white dotted line marks the boundaries between control and LSR-depleted goblet cells (marked by H2B-RFP, pseudo-colored in magenta). **f**, Distribution of normalized F-actin and LSR intensities in control (in green, total n=1119 vertices, 9 embryos, N=4 experiments) and LSR-depleted cells using LSRMO#1 (in magenta, total n=471 vertices, 9 embryos, N=4 experiments). Boxes extend from the 25th to 75th percentiles, with a line at the median and whiskers representing range within 1.5 interquartile range. Two-tailed Mann-Whitney test with 0.05 significance level *1 p=3.31E-56 *2 p=1.02E-177. **g,** LSR-depleted cell (LSR MO#2, marked with H2B-RFP, pseudo-colored in magenta) expressing LifeAct-GFP (pseudo-colored in green) fails to integrate (marked by white arrowhead). A control MCC is marked by a yellow arrowhead. **h,** Quantification of integration success rates. LSR-depleted MCCs are either blocked or disappear (nWT= 48 cells, nLSRMO#2 = 22 cells from 8 embryos, N=5 experiments)

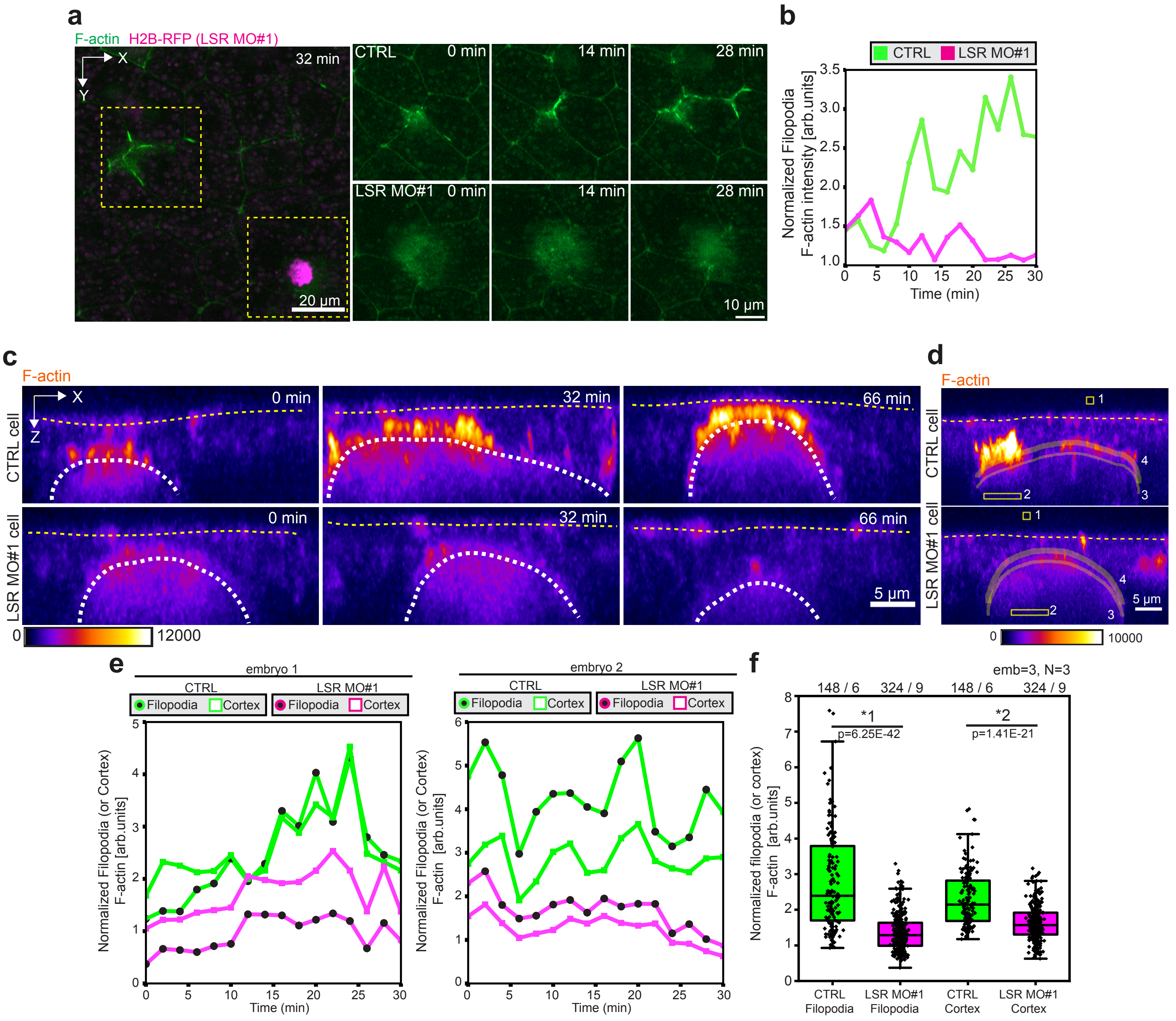

a
F-actin H2B-RFP (LSR MO#1)
X
Y
32 min
20 µm
CTRL 0 min
14 min
28 min
LSR MO#1 0 min
14 min
28 min
10 µm
b
CTRL LSR MO#1
Normalized Filopodia F-actin intensity [arb.units]
Time (min)
c
F-actin
X
Z
CTRL cell
LSR MO#1 cell
0 min
32 min
66 min
5 µm
0 12000
d
F-actin
CTRL cell
LSR MO#1 cell
1
2
3
4
5 µm
0 10000
e
embryo 1
embryo 2
CTRL
LSR MO#1
Filopodia Cortex
Normalized Filopodia (or Cortex) F-actin [arb.units]
Time (min)
f
emb=3, N=3
148 / 6
324 / 9
*1
p=6.25E-42
*2
p=1.41E-21
Normalized filopodia (or cortex) F-actin [arb.units]
CTRL Filopodia
LSR MO#1 Filopodia
CTRL Cortex
LSR MO#1 Cortex

Supplementary Fig. 4 LSR controls F-actin dynamics in MCCs. a,c,d, Control and LSR-depleted cells (LSR MO#1, marked with H2B-RFP, pseudo-colored in magenta) expressing LifeAct-GFP (pseudo-colored in green and as fire as a separate channel). **a,** F-actin dynamics in control and LSR-depleted MCCs. Scale bar: 20 µm. Yellow boxes mark insets for control and LSR-depleted MCC. Scale bar for insets: 10 µm. **b,** Quantification of F-actin accumulation at the filopodia normalized to the trailing edge of control (green) and LSR-depleted (LSR MO#1, magenta) MCCs from a (See Supplementary Fig. 4d). **c,** Orthogonal (XZ) projections used for quantitative analysis of F-actin dynamics in control and LSR-depleted MCCs expressing LifeAct-GFP (pseudo-colored in fire). Mosaic cells were extracted from the same embryo and image gray values were adjusted to the same maximum and minima for visual comparison. **d,** Representation of the ROIs extracted for quantitative analysis: 1 - background, 2 - trailing edge, 3 - cortex and 4 - filopodia. **e,** F-actin intensity at the cortex (squares) and filopodia (black circles) normalized to the trailing edge in control (green) and LSR-depleted cells (magenta) **f,** Box plots representing distribution of normalized F-actin intensities at the cortex and filopodia of control (n=148 timepoints pooled from 6 cells from 3 embryos, N=3 experiments) and LSR-depleted MCCs (n=324 timepoints pooled from 9 cells from 3 embryos, N=3 experiments). Boxes extend from the 25th to 75th percentiles, with a line at the median and whiskers representing range within 1.5 interquartile range. Two-tailed Mann-Whitney test with a 0.05 significance level *1 p=6.25E-42 *2 p=1.41E-21.

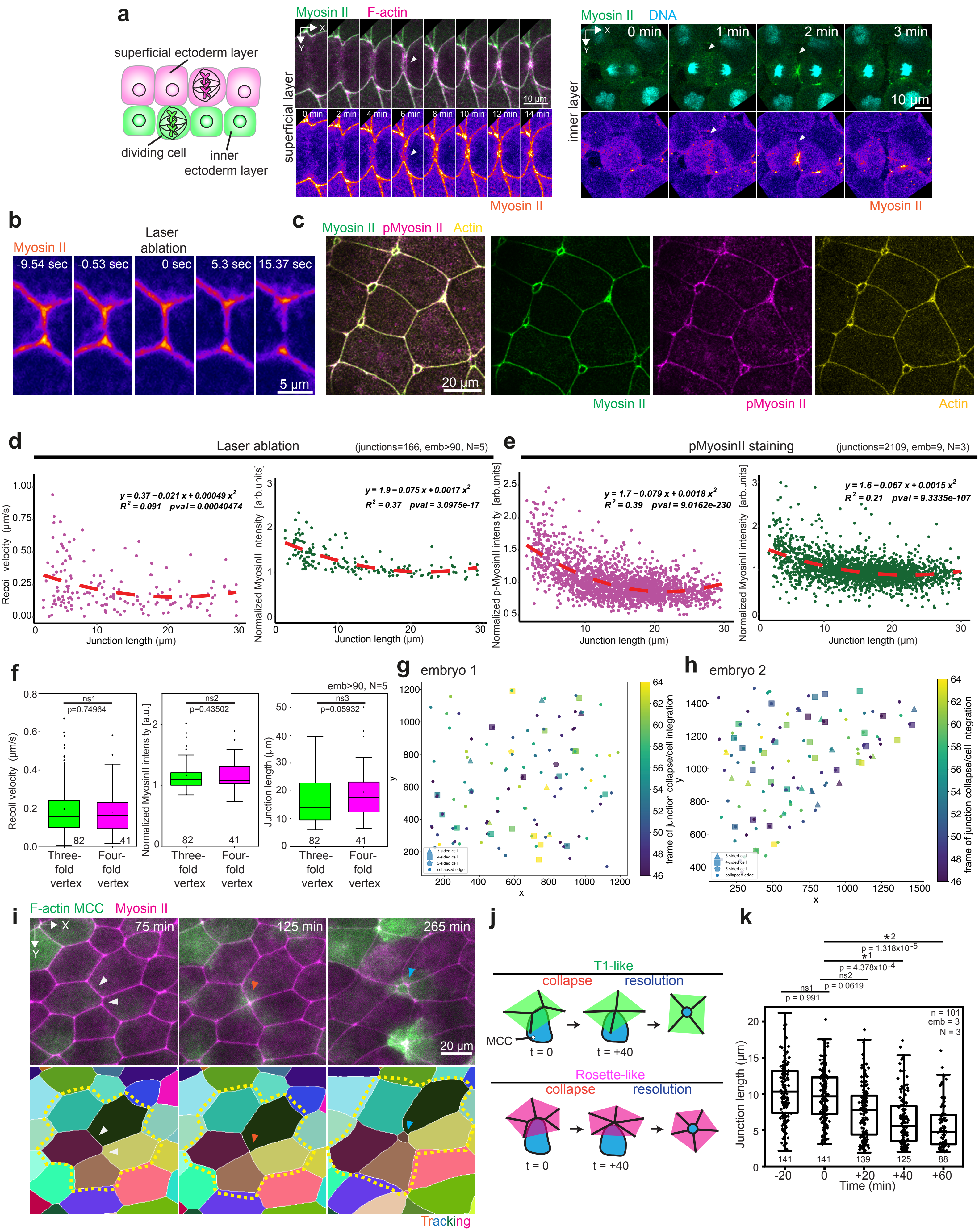
a
superficial ectoderm layer
dividing cell
inner
ectoderm layer
Myosin II F-actin
X
Y
superficial layer
0 min
2 min
4 min
6 min
8 min
10 min
12 min
14 min
10 μm
Myosin II
Myosin II DNA
0 min
1 min
2 min
3 min
inner layer
10 μm
Myosin II
b
Myosin II
Laser
ablation
-9.54 sec
-0.53 sec
0 sec
5.3 sec
15.37 sec
5 μm
c
Myosin II pMyosin II Actin
20 μm
Myosin II
pMyosin II
Actin
d
Laser ablation
(junctions=166, emb>90, N=5)
y = 0.37 − 0.021 x + 0.00049 x²
R² = 0.091 pval = 0.00040474
Recoil velocity (μm/s)
Junction length (μm)
y = 1.9 − 0.075 x + 0.0017 x²
R² = 0.37 pval = 3.0975e-17
Normalized MyosinII intensity [arb.units]
Junction length (μm)
e
pMyosinII staining
(junctions=2109, emb=9, N=3)
y = 1.7 − 0.079 x + 0.0018 x²
R² = 0.39 pval = 9.0162e-230
Normalized p-MyosinII intensity [arb.units]
Junction length (μm)
y = 1.6 − 0.067 x + 0.0015 x²
R² = 0.21 pval = 9.3335e-107
Normalized MyosinII intensity [arb.units]
Junction length (μm)
f
ns1
p=0.74964
Recoil velocity (μm/s)
82
41
Three-fold vertex
Four-fold vertex
ns2
p=0.43502
Normalized MyosinII intensity [a.u.]
82
41
Three-fold vertex
Four-fold vertex
emb>90, N=5
ns3
p=0.05932
Junction length (μm)
82
41
Three-fold vertex
Four-fold vertex
g
embryo 1
3-sided cell
4-sided cell
5-sided cell
collapsed edge
x
y
frame of junction collapse/cell integration
h
embryo 2
3-sided cell
4-sided cell
5-sided cell
collapsed edge
x
y
frame of junction collapse/cell integration
i
F-actin MCC Myosin II
X
Y
75 min
125 min
265 min
20 μm
Tracking
j
T1-like
collapse
resolution
MCC
t = 0
t = +40
Rosette-like
collapse
resolution
t = 0
t = +40
k
*2
p = 1.318x10⁻⁵
*1
p = 4.378x10⁻⁴
ns2
p = 0.0619
ns1
p = 0.991
n = 101
emb = 3
N = 3
Junction length (μm)
141
141
139
125
88
-20
0
+20
+40
+60
Time (min)

Supplementary Fig. 5: Validation of myosin II sensor and vertex model predictions. a-e, The SF9-3xGFP myosin II sensor serves as a proxy for junctional tension. **a,** The SF9-3xGFP sensor (myosin II) localizes to the cleavage furrow of superficial (left panel) and inner (right panel) dividing ectodermal cells. **b,** Laser ablation of superficial epithelial cell junctions. Myosin II is pseudo-colored in fire. **c,** Phosphomyosin-II staining of superficial epithelium expressing the SF9-3xGFP myosin II sensor. SF9-3xGFP myosin II sensor (myosin II) is pseudo-coloured in green, active phosphorylated myosin (pmyosin II) is pseudo-coloured in magenta and F-actin is pseudo-coloured in yellow. **d,** Analysis of laser ablation data. For recoil data: equation ($y=0.37-0.021x+0.0004074x^2$), goodness of fit ($R2=0.091$) and two-sided F-test p-value (0.0004074). For myosin II: equation ($y=1.9-0.075x+0.0017x^2$), goodness of fit ($R2=0.37$) and two-sided F-test p-value (3.0975e-17) (junctions=166 from >90 embryos, N=5 experiments). **e,** Analysis of phosphomyosin II (pmyosin II) and SF9-3xGFP (myosin II) staining data. For pmyosinII data: equation ($y=1.7-0.079x+0.0018x^2$) , goodness of fit ($R2=0.39$) and F-test p-value (9.0162e-203). For myosin II: equation ($y=1.6-0.067x+0.0015x^2$) , goodness of fit ($R2=0.21$) and F-test p-value (9.3335e-107) (junctions=2109 from 9 embryos, N=3 experiments). **f,** Comparison of recoil velocities, myosin II intensities and length between junctions connected to either three-fold vertex or four-fold vertex (82 three-fold vertices and 41 four-fold vertices from >90 embryos, N=5 experiments). Boxes extend from the 25th to 75th percentiles, with a line at the median and whiskers representing range within 1.5 interquartile range. Two-tailed Mann-Whitney test with 0.05 significance level. ns1 p=0.74964, ns2 p=0.43502, ns3 p=0.05932. **g-h,** Spatial correlation between epithelial junction collapse (circles) and MCC integration (polygons), colored by the time of occurrence for two different embryos. **i,** Example of a higher-fold MCC integration. Top panel, snapshots of MCC integrating in a higher-order vertex. MCC is labeled with α-tubulin::LifeAct-RFP (pseudo-colored in green) and the myosin intrabody with SF9-3xGFP (pseudo-colored in magenta). Bottom panel, Segmented image. White arrowhead mark initial epithelial junction layout, orange arrowhead mark junction collapse and cyan arrowhead marks apical expansion. The yellow outline depicts the formation of a rosette-like structure. Scale bar: 20 µm. **j,** Schematics of cell integration at higher-fold vertices, which is divided in a remodeling phase and a resolution phase. Integration at a 4-fold vertex resembles a T1 event (T1-like) whereas 5-fold integration or higher resembles the formation of a rosette (Rosette-like). **k,** Epithelial junction length distribution involved in formation of higher-order vertices. T=0 marks the onset of MCC reaching the overlying junction (number of junctions is specified for each timepoint, n=101 cells from 3 embryos, N=3 experiments). Boxes extend from the 25th to 75th percentiles, with a line at the median and whiskers representing range within 1.5 interquartile range. One-way Analysis of Variances (ANOVA) with Tukey's test and a significance level of 0.05 was used to compare difference junction lengths with the reference junction length (junction length at the onset of MCC integration, T=0). ns1 p=0.991, ns2 p=0.0619, *1 p= 4.378E-4, *2 p=1.318E-5.

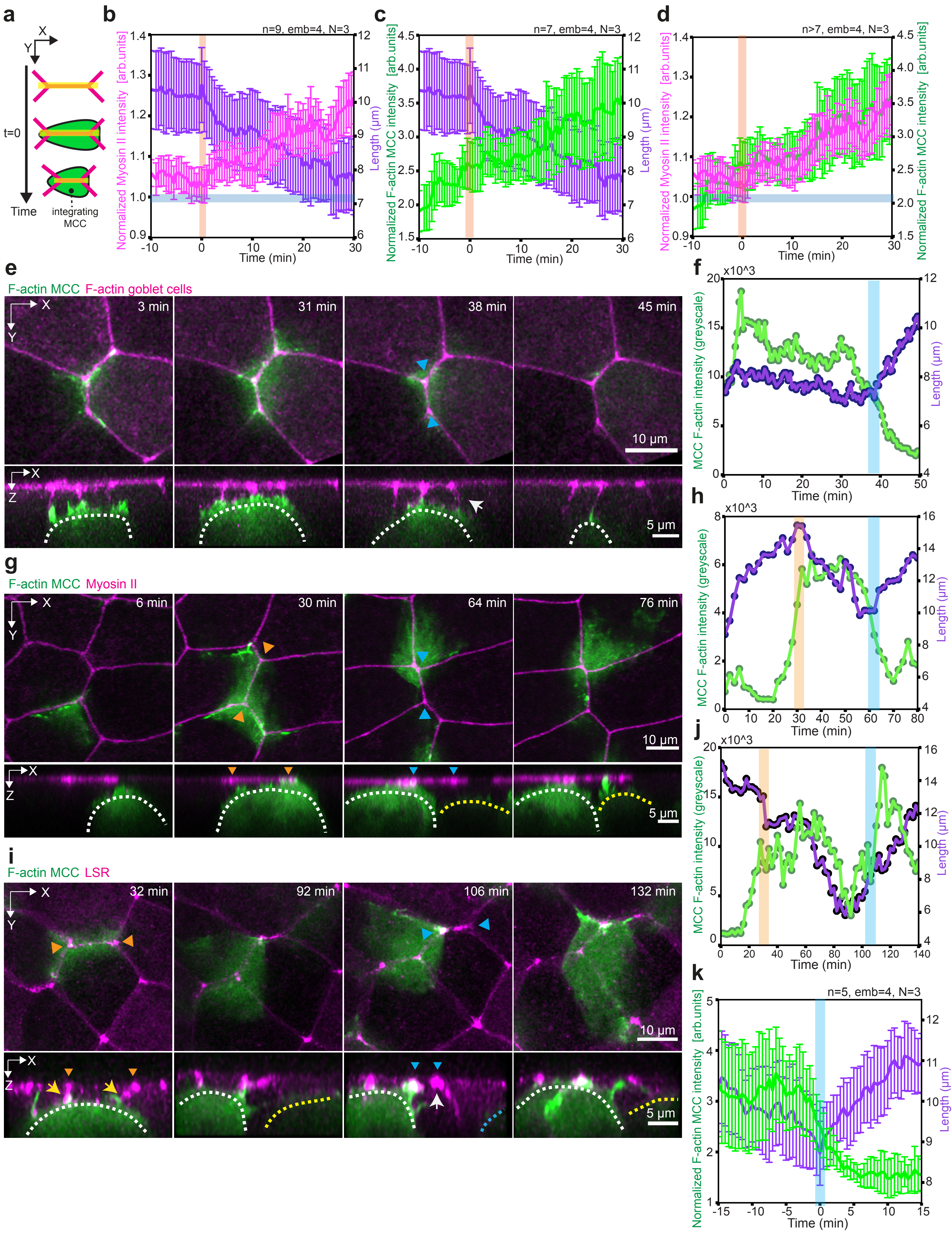
a
b
c
d
X
Y
t=0
Time
integrating MCC
n=9, emb=4, N=3
n=7, emb=4, N=3
n>7, emb=4, N=3
Normalized Myosin II intensity [arb.units]
Normalized F-actin MCC intensity [arb.units]
Length (µm)
Time (min)
e
F-actin MCC F-actin goblet cells
3 min
31 min
38 min
45 min
10 µm
5 µm
Z
f
x10^3
MCC F-actin intensity (greyscale)
g
F-actin MCC Myosin II
6 min
30 min
64 min
76 min
h
i
F-actin MCC LSR
32 min
92 min
106 min
132 min
j
k
n=5, emb=4, N=3

Supplementary Fig. 6 MCCs pull on overlying epithelial vertices to induce junction collapse. a-k, Orange vertical bars and arrowheads mark the onset of epithelial junction collapse, cyan vertical bars and arrowheads mark the onset of junction retraction. **a,** Schematics representing epithelial junction (in magenta) collapse quantification during MCC (in green) integration. t=0 indicates the start of epithelial junction collapse, which coincides with MCC reaching the overlying junction. **b,** Average normalized junctional myosin-II intensity (magenta) and junction length (purple) before and during junction collapse (t=0, the start of junction collapse)(n=9 junctions from 4 embryos, N=3 experiments). **c,** Average normalized MCC F-actin intensity (green) and junction length (purple) before and during junction collapse (t=0, the start of junction collapse)(n=7 junctions from 4 embryos, N=3 experiments). **d,** Normalized junctional myosin-II intensity (magenta) and normalized MCC F-actin intensity (green) during junction collapse (t=0, the start of junction collapse)(n>7 junctions from 4 embryos, N=3 experiments). **e,g,i,** Image sequence depicting epithelial junction retraction after MCC (pseudo colored in green) loses contact (marked by cyan arrowheads) with the vertex. Junctions are labeled with nectin Utrophin-RFP in e, SF9-3xGFP in g and LSR-3xGFP in i. White dotted line marks the cell of interest, the yellow dotted line marks the competing MCC. The yellow and white arrows mark contact formation and loss with the vertex, respectively. **f,h,j,** Corresponding MCC F-actin intensity in grey values (green) and junction length (purple) during junction retraction. **k,** Average normalized MCC F-actin intensity (green) and junction length (purple) before and after retraction (marked by t=0)(n=5 junctions from 4 embryos, N=3 experiments). **b-d, k,** data show mean ± SEM.

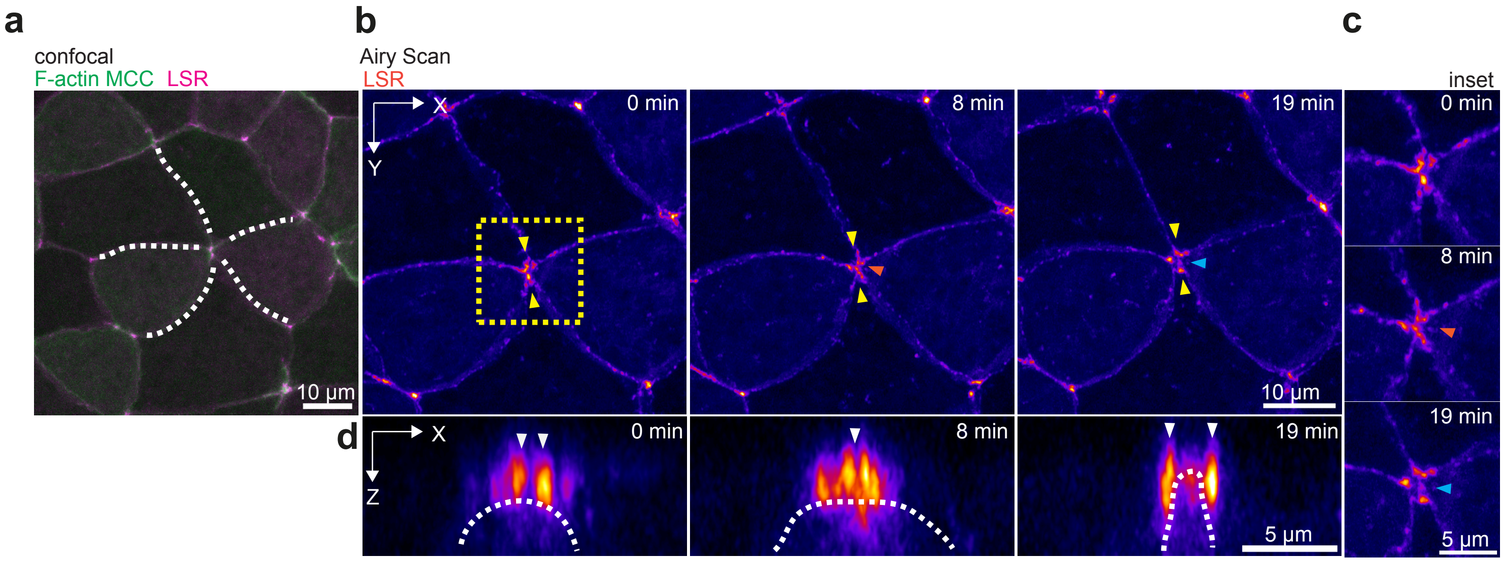
a
confocal
F-actin MCC LSR
10 µm
b
Airy Scan
LSR
X
Y
0 min
8 min
19 min
10 µm
c
inset
0 min
8 min
19 min
5 µm
d
X
Z
0 min
8 min
19 min
5 µm

Supplementary Fig. 7: Live Superresolution Imaging of Rosette formation. Rosette formation during MCC integration. **a,** Confocal image of a rosette formation event which was then imaged using the Superresolution mode of the Airy Scan module (Airy Scan). **b-d,** Airy Scan imaging of rosette formation. Orange arrowheads marks formation of multi-fold vertex and blue arrowheads marks initiation of apical expansion. **b,** Epithelial vertices can be distinguished before (timepoint 0 min) and after MCC integration (timepoint 19 min). During integration (timepoint 8 min) the LSR strings cannot be easily distinguished from the LSR accumulated at the leading edge of the MCC. Yellow box outlines close-up in c and white arrowheads mark orientation of orthogonal projections in d. **c,** Close-up on rosette formation. **d,** Orthogonal (XZ) projections of rosette formation during MCC integration. White arrowheads mark vertex position.

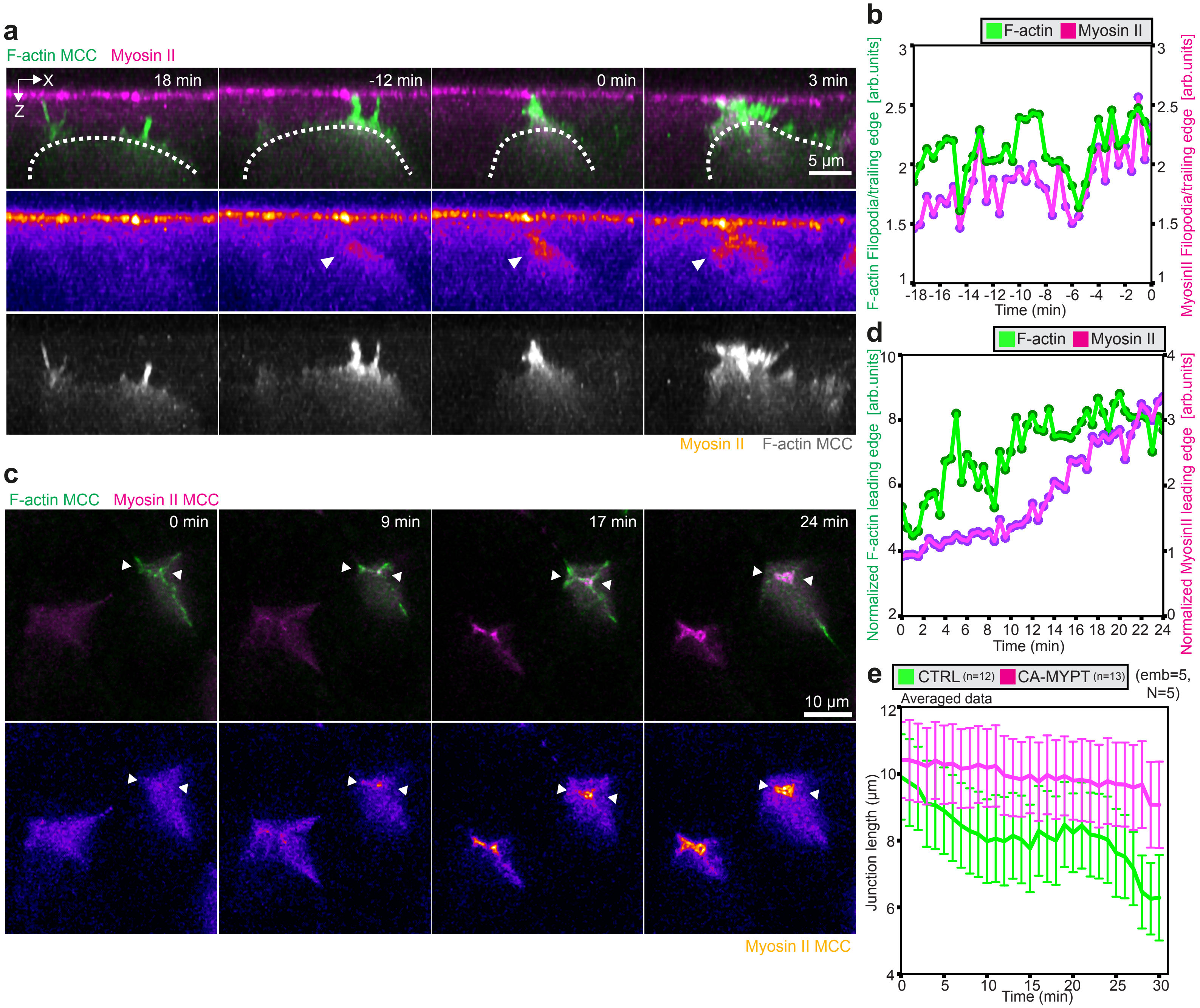
a
F-actin MCC
Myosin II
X
Z
18 min
-12 min
0 min
3 min
5 µm
Myosin II
F-actin MCC
b
F-actin
Myosin II
F-actin Filopodia/trailing edge [arb.units]
MyosinII Filopodia/trailing edge [arb.units]
Time (min)
c
F-actin MCC
Myosin II MCC
0 min
9 min
17 min
24 min
10 µm
Myosin II MCC
d
F-actin
Myosin II
Normalized F-actin leading edge [arb.units]
Normalized MyosinII leading edge [arb.units]
Time (min)
e
CTRL (n=12)
CA-MYPT (n=13)
(emb=5, N=5)
Averaged data
Junction length (µm)
Time (min)

Supplementary Fig. 8 Myosin II is recruited to the leading edge of MCCs during probing and junction remodeling. a, Orthogonal (XZ) projections of myosin II recruitment to the MCC leading edge during integration. MCC is labeled with α-tubulin::LifeAct-RFP (pseudo colored green in a composite image, pseudo colored in gray as a single channel) and myosin II is labeled with myosin intrabody SF9-3xGFP (pseudo colored magenta in the composite image and pseudo colored in fire as a single channel). Scale bar: 5 µm. t=0 marks the last tracked frame during integration. **b,** Normalized myosin-II intensity (magenta) and normalized MCC F-actin intensity (green) at the leading edge of integrating MCC. (Also see Fig. 5b) **c,** Image sequence depicting myosin recruitment in MCC during junction remodeling. White arrowheads depict myosin accumulation. MCC is labeled with α-tubulin::LifeAct-RFP (pseudo colored green in a composite image, pseudo colored in gray as a single channel) and myosin II is labeled with myosin intrabody SF9-3xGFP (pseudo colored magenta in the composite image and pseudo colored in fire as a single channel). **d,** Normalized myosin II intensity (magenta) and normalized MCC F-actin intensity (green) at the leading edge of inserting MCC. **e,** Average junction length for control and CAMYPT-overexpressing MCCs after MCCs reached the overlying junctions, t=0 (nWT= 12 junctions, nCAMYPT = 13 junctions, N=5 experiments). Data show mean ± SEM.

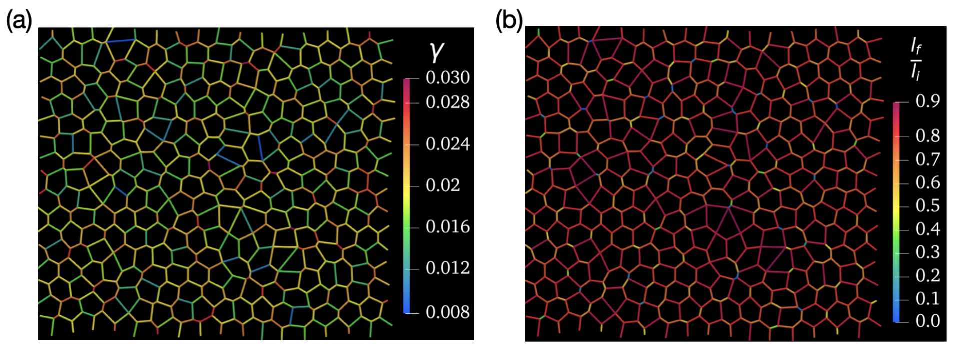

Supplementary Figure 9. Edge collapse due to line tension perturbations. (a) Initial configuration is defined as force-balanced tissue configuration with heterogeneous line tensions with average $<\gamma>= 0.02$. **(b)** The line tension of each edge i is perturbed to γ_i+0.2$<\gamma>$. Then we find the new force balance configuration and by calculating the ratio of final edge length to initial length before perturbation l_f/l_i, we find that bonds with higher line tension and shorter length are more likely to collapse (see Supplementary Note 1).

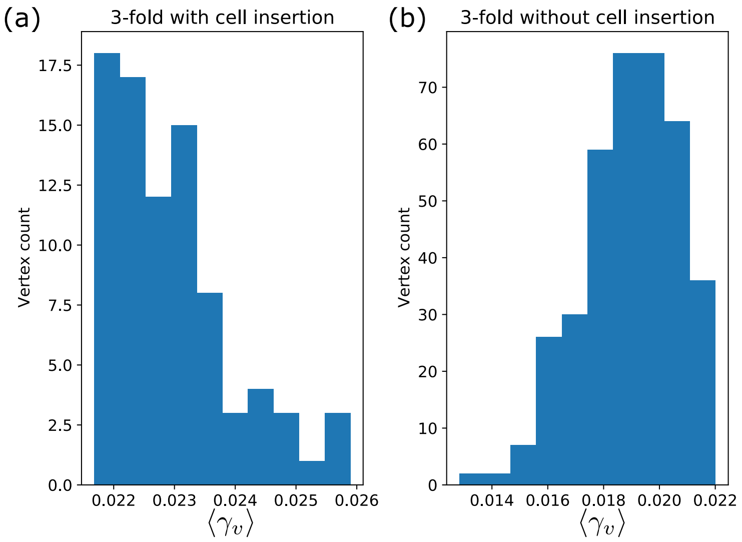

Supplementary Figure 10. Number of 3-fold vertices in the tissue with successful **(a)** and failed **(b)** MCC integration, with respect to the average tension $\langle\gamma\rangle$ of bonds abutting at those vertices (see Supplementary Note 1).

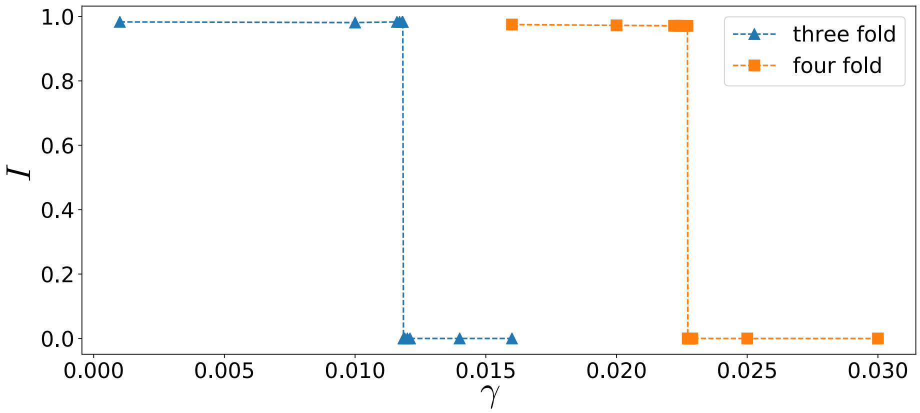

Supplementary Figure 11. Comparing the line tension criteria for MCC intercalation by computing the order parameter I, defined as the ratio of area of intercalated cell at force balance to the cell preferred area, for three-fold (blue) and four-fold (orange) vertices. The calculations are done in a hexagonal cellular network with a uniform line distribution (see Supplementary Note 1).

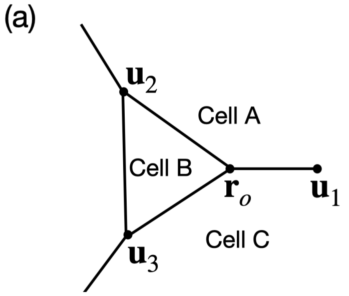

Supplementary Figure 12. Schematics of symmetric insertion of cell B at a three-fold vertex **(a)**, and at a four-fold vertex **(b)** at location $\mathbf{r}_o$. Upon integration, each vertex of cell B is subject to forces stemming from the line tension of the 3 connected bonds in directions $\mathbf{u}_1 - \mathbf{r}_o$, $\mathbf{u}_2 - \mathbf{r}_o$ and $\mathbf{u}_3 - \mathbf{r}_o$ and the pressure difference between cell B and its neighboring cells A and C (see Supplementary Note 1).

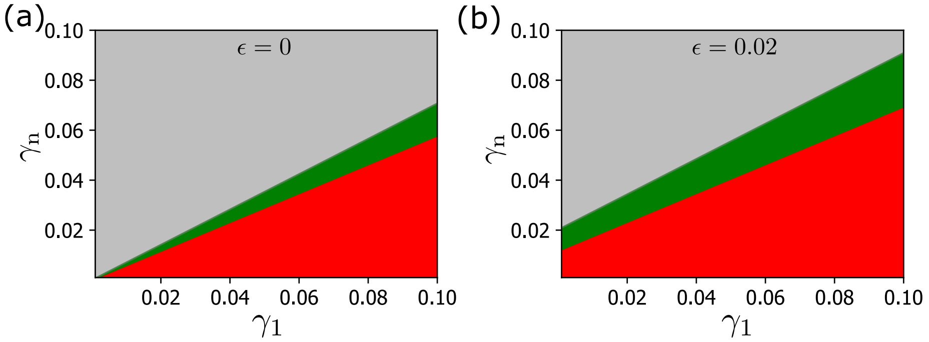

Supplementary Figure 13. Stability diagram for the intercalation at 3-fold and 4-fold vertices. Gray: neither three-fold nor four-fold allows a cell intercalation. Green: Only four-fold vertices allow cell intercalation. Red: Both three-fold and four-fold vertices allow for cell intercalation. The diagram is given for both Infinitesimal size **(a)** and finite size of inserting cells **(b)**. (see Supplementary Note 1)

Supplementary Table 1

Reagent or Resource	Source	Identifier
**Chemicals**		
Ultra-Low Melting Point Agarose	Sigma Aldrich	A2576-5G
Abberior Start Phalloidin-488	Abberior	
Alexa647 Phalloidin	Invitrogen	A22287
CasBlock™	ThermoFisher	008120
Fetal Bovine Serum	Sigma Aldrich	F2442-100ML
Bovine Serum Albumin	Sigma Aldrich	AM2616
**Antibodies**		
Anti-*Xenopus* Angulin1/LSR	Higashi et al., 2016	
Anti-phospho Myosin Light Chain 2	Cell Signaling	3671S
Donkey Anti-rabbit Alexa647	ThermoFisher	A-31573
Donkey Anti-rabbit Cy3	Jackson Immunoresearch	112-166-143
**Oligonucleotides**		
Primers for cloning angulin-1 in α-tubulin backbone:<br><br>Fwd: caccATGGAAGGGACCGGAATTG<br>Rev: AACGACCAAACTCTCTCGGC		

Primers for cloning angulin-1 in nectin backbone:<br><br>Fwd:<br>aacggtGGATCCATGGAAG GGACCGGAATTG<br>Rev:<br>aacggtGCGGCCGCAAAAA ACCTCCCACACCTCCC		
Primers for cloning SF9 in pCS2+ 3xGFP backbone:<br><br>Fwd:<br>aacggtATCGATATGGCCGA GGTGCAGCTGG<br>Rev:<br>aacggtTCTAGAACCTAGGA CGGTCAGCTTGGTC		
**Recombinant DNA**		
DNA probes for cell specific expression		
pαtubulin::LifeAct-GFP	Sedzinski et al., 2016	
pαtubulin::LifeAct-RFP	Sedzinski et al., 2016	
pαtubulin::LSR-GFP	This study	
pnectin::Utrophin-RFP	Sedzinski et al., 2016	
pnectin::LSR-GFP	This study	
pnectin::LSR-RFP	This study	
Plasmids used for mRNA synthesis		
pCS2+/LifeAct-GFP	Wallingford Lab	
pCS2+/H2B-RFP	Wallingford Lab	

pCS2+/LSR-3xGFP	Higashi et al., 2016	
pCS2+/SF9-3xGFP	This study	
pCS2+/CA-MYPT	Barriga et al., 2019	
**Oligomorpholinos (MO)**		
Transcription blocker LSR morpholino (LSR MO#1): gagaacatggaagggaccggaattg	Gene Tools USA	
Splice blocker LSR morpholino (LSR MO#2): ggccgatcctaagggaataagaaga	Gene Tools USA	

Supplementary Table 2

**Figure #**	# of experiments	# of embryos	# of cells	# of events	Notes
1b and 1d	10	10	18		
2a - 2c	4	4	17		
2d	2	2	5		
2e	3	3	4	9 pulling events	
4a	3	3			
6a	3	4	9		
6f	3	4	5		
7a	3	3	10		
7c	6	6	55 WT cells and 24 CA-MYPT cells		
7e	5	5	13		
**Supplementary Figure #**	# of experiments	# of embryos	# of cells	# of events	
1a	1	1	1		
1b and 1c	10	10	18		Same as figure 1b and 1d
2a and 2b	3	3	4	9 pulling events	Same as figure 2e
2d	6	6	15		
2e	2	2			
2f	9	9	18		
2g	1	1	1		
2h	2	2	2		
3a	5	5	26 WT cells and 22 LSRMO#1		Same as supplementary figure 3b
3c	5	5	6		
3d	3	3	3		
3e	4	9			Same as supplementary

					figure 3f
3g	4	8	49 WT cells and 22 LSRMO#2		Same as supplementary figure 3h
4a	3	3	6 WT cells and 9 LSRMO#1 cells		
4c	3	3	6 WT cells and 9 LSRMO#1 cells		
5a	1	1			
5b	5	90		166 junctions	
5c	3	9			
5i	3	3		195 events	
6e	1	1	1		
6g	1	1	1		
6i	1	1	1		
7a-d	3	3	3		
8a	3	3	10		Same as figure 6b
8c	2	2	3		

SUPPLEMENTARY NOTE 1: THEORETICAL MODEL

In this work, we construct a two-dimensional vertex model to provide a mechanistic understanding of the process of probing and inserting by Multiciliated cells (MCCs) on the overlaying epithelium. This choice is motivated by the simplicity of this class of models that involves small set of control parameters, while providing proper account of cellular shape changes together with edge and vertex dynamics [1]. To construct the vertex model of a monolayer of cells a work functional is defined as

$$W = \frac{1}{2}\sum_{\alpha} K^{2\mathrm{D}} \left(A_\alpha - A_0\right)^2 + \sum_i \Gamma_i L_i \quad , \tag{S.1}$$

where indices α and i run over each cell and each edge of the cellular network, respectively. The parameter Γ_i is the line tension within an edge i with length L_i. Here for simplicity, we assume all cells have identical target area A_0 and area stiffness $K^{2\mathrm{D}}$. In case of homogeneous line tension Γ within the tissue, the mechanical properties of the cells are controlled by a single dimensionless parameter $\gamma = \Gamma/(K^{2\mathrm{D}} A_0^{3/2})$. This definition of the work function given in Eq. (S.1), corresponds to a network with positive shear modulus for $\gamma > 0$, and leads to a soft network for $\gamma \leq 0$ where the ground state is degenerate under a shear deformation. In this study, we focus on the solid regime $\gamma > 0$. Having defined the work functional Eq. (S.1), we compute the force on each vertex $\mathbf{F}_v = -\partial W/\partial \mathbf{X}_v$ and evolve the vertex position following the dynamical equation:

$$\frac{d\mathbf{X}_v}{dt} = \mu \mathbf{F}_v \quad , \tag{S.2}$$

where μ is the mobility coefficient. To find the minimum energy configuration, not only the vertices move down the gradient according to Eq. (S.2), but also tissue network undergoes T1 topological transitions that are energy favorable. At each time step of the simulation we randomly pick a short edge (with length smaller than ε) and check the energy change as a result of flipping this edge ΔW_{T1}. We only accept a T1 transition when $\Delta W_{T1} < 0$. Since we are interested in the force balance tissue configuration, we continue our simulation steps until the energy difference between two consecutive time steps is less than $f_{\mathrm{tol}} = 10^{-14}$.

Vertex probing by intercalating cells. Experimental results indicate the existence of vertices with varying number of connecting edges, predominantly those with three edges (3-fold vertex) and four edges (4-fold vertex) connecting to them. To this end, we begin by simulating a monolayer of cells with heterogeneous line tension. We choose line tensions at the cell bonds within the tissue randomly from a normal distribution with the mean value of γ and the standard deviation of $\gamma/4$. The choice of variable line tension at the cell edges ensures that both stable 3-fold and 4-fold vertices can form within the monolayer (see Ref. [2]).

Supplementary Fig. 9a shows a typical configuration of cells as a result of simulating the vertex model (Eqs. S.1-S.2). The formation of heterogeneous cell shapes with both 3-fold and 4-fold stable vertices that are connected via edges of different line tension is evident. Our goal is to understand which vertices are more susceptible to be opened up by intercalating cells.

Our experimental observations demonstrate that before inserting themselves, the intercalating cells probe different vertices by extending their filopodia, making contact with the goblet cells in the monolayer, and pulling on them. In some cases this pulling continues until the intercalating cell opens up the vertex and insets itself within the monolayer, while in some other instances the intercalating cell moves to another vertex after the initial probing (see main text). As such we conjecture that the intercalating MCCs have the ability of probing local properties of the monolayer before starting to insert themselves at particular locations. To test this, we introduce a probing mechanism to the vertex model: at each vertex an out-of-plane force of a fixed magnitude f is applied, while fixing all other vertices in plane, and the out-of-plane displacement δ of the probe vertex as a result of the applied force is measured. The local measure of the stiffness at each vertex is then obtained as $\kappa_\delta = f/\delta$. Repeating this procedure for all the vertices in the monolayer gives the map of local stiffness throughout the monolayers (Fig. 3b in main text). It is clear from the stiffness map that this mechanism of out-of-plane pulling effectively probes the local tensions around different vertices distinguishing vertices with connecting edges of high line tension from those that have low tension connectivity. This is interesting, because it suggests that the pulling mechanism adapted by MCCs could act as an effective probing of the local tension within the monolayer. Next we ask if there is any correlation between this local probing and the preferable locations of cell insertion.

Mechanism of vertex selection for intercalation. In order to explore the propensity of different vertices to intercalation, we introduce an inserting cell at each of the vertices, one at a time. The inserting cell is added with an initial area much smaller than the target area of the cell $A_{in} \ll A_0$. As such the pressure difference between the inserting cell and its neighbors works to expand the cell towards its target area. This is resisted by the cell's

line tension on one hand, and is enhanced by the tension from surrounding edges at the other. As a result of this competition the inserting cell either grows to its final area or shrinks and disappears at the vertex. We distinguish between two different scenarios by defining a binary order parameter that specifies whether the cell intercalation at the vertex is successful and the MCC can integrate into the monolayer or not. Calculating the intercalation order parameter at each vertex and comparing with the probing results from the previous section we clearly observe the propensity of successful intercalation at the points where the local stiffness κ_δ is highest (Fig. 3b-c in main text). This is because relatively larger line tension from surrounding edges enhances the expansion of the inserting cell into the monolayer. Interestingly, we also observe that while all 4-fold vertices open up, only a fraction of 3-fold vertices with high local stiffness are chosen for intercalation. This can be further quantified by calculating the histogram of the number of 3-fold vertices that open up with respect to their local average tension. Remarkably, with increasing the line tension of the inserting cells less and less 3-fold vertices are able to open up, while still all 4-fold vertices lead to an intercalation event (Supplementary Fig. 10). These results indicate that in an heterogeneous tissue a 4-fold vertex is more susceptible to be opened up by the inserting cells and as such 4-fold vertices are potential hotspots for intercalation. To understand the underlying mechanism for this distinction we next take a closer look at the instability of 3-fold and 4-fold with respect to an intercalation event.

Criteria for vertex opening. In order to pinpoint the mechanistic basis of the higher intercalation probability at 4-fold vertices compared to the 3-fold vertices, here we consider a simplified setup of one intercalating cell within a pre-imposed configuration of cells with either 3-fold or 4-fold vertices. Moreover, we consider uniform constant tension across the monolayer and choose the same value of tension for the inserting cell such that the only difference between the 3-fold and 4-fold vertex is their configuration. We then numerically explore the possibility of intercalation for various values of tension. The results show a striking difference between the 3-fold and 4-fold vertex, demonstrating that for identical situations a 4-fold vertex opens up at a threshold value that is smaller than a 3-fold vertex (Supplementary Fig. 11). To better understand this process, we next analytically investigate the criteria for vertex opening and compare our prdiction with the simplified setup discussed above.

Here we explore the intercalation of cells into the cellular network of an epithelium defined by our vertex model. We assume that the tissue can consist of three-fold, four-fold and higher fold cellular junctures. We compare the criteria for a successful cell insertion at the location of vertices with different number of connected edges.

As is depicted in Supplementary Fig. 12, cells inserted at three-fold and four-fold vertices are triangular and quadrilateral respectively, and by extension n-gon for n-fold vertices. After a cell insertion, we calculate the initial forces imposed on the vertices of the inserted cells and find the criteria with which these forces are in the outward direction from the inserted cell, a necessary condition for a successful cell intercalation at the vertex.

The net force imposed on the vertex $\mathbf{r}_o$ is given by:

$$\mathbf{F}_{\mathbf{r}_o} = \frac{P_A}{2}\left[\hat{z} \times (\mathbf{u}_2 - \mathbf{u}_1)\right] + \frac{P_B}{2}\left[\hat{z} \times (\mathbf{u}_3 - \mathbf{u}_2)\right] + \frac{P_C}{2}\left[\hat{z} \times (\mathbf{u}_1 - \mathbf{u}_3)\right] \tag{S.3}$$

Without loss of generality we assume the vertex $\mathbf{r}_o$ at the origin of the reference frame, and $\mathbf{u}_1 - \mathbf{r}_o$ along the x-axis. Thus the opening force can be computed by projecting the net force along $\mathbf{u}_1$:

$$\mathbf{F}_{\mathbf{r}_o} \cdot \hat{u}_1 = \Gamma_1 + \Gamma_2 \hat{u}_1 \cdot \hat{u}_2 + \Gamma_3 \hat{u}_1 \cdot \hat{u}_3 + \frac{P_A}{2}\hat{z} \cdot (\mathbf{u}_2 \times \hat{u}_1) + \frac{P_B}{2}\hat{z} \cdot [\hat{u}_1 \times (\mathbf{u}_2 - \mathbf{u}_3)] + \frac{P_C}{2}\hat{z} \cdot (\hat{u}_1 \times \mathbf{u}_3) \tag{S.4}$$

Given that $\measuredangle \mathbf{u}_1 \mathbf{r}_o \mathbf{u}_2 = \alpha$ and $\measuredangle \mathbf{u}_1 \mathbf{r}_o \mathbf{u}_3 = \beta$, we can re-write Eq. (S.4) in terms of angles between the incident edges at $\mathbf{r}_o$:

$$\mathbf{F}_{\mathbf{r}_o} \cdot \hat{u}_1 = \Gamma_1 + \Gamma_2 \cos\alpha + \Gamma_3 \cos\beta + \frac{1}{2}(P_B - P_A)|\mathbf{u}_2| \sin\alpha + \frac{1}{2}(P_C - P_B)|\mathbf{u}_3| \sin\beta \tag{S.5}$$

The necessary condition for a successful cell insertion reads:

$$\mathbf{F}_{\mathbf{r}_o} \cdot \hat{u}_1 > 0. \tag{S.6}$$

To simplify this criteria, we consider completely symmetric configuration where the n-fold vertex opens into a regular n-gon (i.e. the three-fold vertex opens into an equilateral triangle and the four-fold vertex opens to a square).

Furthermore, by assuming that cells A and C have the same pressure equal to P_N, and defining the pressure of cell i as $P_i = -K^{2\mathrm{D}}(A_i - A_0)$, the opening force on an n-fold vertex simplifies to:

$$\mathbf{F}_{\mathbf{r}_o}.\hat{u}_1 = \Gamma_1 - (\Gamma_2 + \Gamma_3)\sin(\frac{\pi}{n}) + \frac{K^{2\mathrm{D}}}{2}(A_N - A_B)\cos(\frac{\pi}{n})(|\mathbf{u}_2| + |\mathbf{u}_3|) \tag{S.7}$$

We can simplify this even further, by assuming $|\mathbf{u}_2| = |\mathbf{u}_3| = \epsilon\sqrt{A_0}$, and equal line tension for edges of the inserted cell $\Gamma_2 = \Gamma_3 = \Gamma_n$. The opening force normalized by line tension $f_o = \mathbf{F}_{\mathbf{r}_o} \cdot \hat{u}_1/\Gamma_1$:

$$f_o = 1 - 2\frac{\gamma_n}{\gamma_1}\sin(\frac{\pi}{n}) + \frac{a_N - a_B}{\gamma_1}\epsilon\cos(\frac{\pi}{n}), \tag{S.8}$$

where the dimensionless quantities are $a_i = A_i/A_0$, and $\gamma_i = \Gamma_i/(K^{2\mathrm{D}}A_0^{3/2})$. For positive values of line tension parameter Γ_1, the opening condition given in Eq. (S.6) reads $f_o > 0$. Supplementary Fig. 13 shows the cell intercalation regimes based on Eq (S.8) only for three-fold and four-fold vertices.

Probing and collapsing edges for intercalation. Not only the intercalating cells prefer inserting themselves at 4-fold vertices rather than 3-fold ones, but also we explore the possibility of them actively rearranging the epithelial layer to form 4-fold vertices. This is possible for example by probing and pulling at two vertices of the same edge and closing that edge such that two adjacent 3-fold vertices collapse into a 4-fold vertex, which we showed in previous section is a preferential cite for the cell to insert itself. To investigate this interesting mechanism, we conduct numerical simulations, in which we probe the response of edges - one at a time - to external contraction. This is informed from the experimental observations that show intercalating MCCs can pull simultaneously on the two vertices of the same edge. We thus conjecture that depending on the length and the tension of the edge, this simultaneous pulling can induce an edge collapse actively remodeling the epithelial layer to form preferential 4-fold vertices. The results of these simulations are depicted in Supplementary Fig. 9b. In order to quantify the edge fate under tensional perturbation, we define l_f/l_i as the order parameter characterising the ratio of the particular edge's length after applying the perturbation to that edge's initial length. As such, if an edge collapses under tensional perturbation and a 4-fold vertex is formed, the order parameter is zero, $l_f/l_i = 0$, and it is finite otherwise. The simulation results show that the edge collapse and the subsequent 4-fold vertex formation can only occur for edges with sufficiently short length and sufficiently large tension. This can be more clearly seen from the stability-diagram of l_f/l_i in the length-tension phase space clearly demonstrating the propensity of edge collapse for small initial length $l_b(t = t_0)$ and high initial tensions $\gamma_b(t = t_0)$ within the edge (see Fig. 4h in the main text). Our experimental observations (Fig. 4i) similarly show that the likelihood of junction (edge) collapse in the tissue's cellular network increases with normalized Myosin II intensity of the junctions and decreases with the normalized length of the junction (Length/$\sqrt{A_{avg}}$), where A_{avg} is defined as the average area of the cells in the epithelium. Taken together, these results show that when intercalating MCCs pull on two vertices of an edge in the goblet layer, they can indeed induce a re-arrangement in the goblet layer by closing that edge and forming a 4-fold vertex, which in turn is a preferential site for the MCCs to insert themselves. Therefore, not only MCCs take advantage of existing 4-fold vertices in the goblet layer, they can even create their own 4-fold vertex from an edge, if the edge is sufficiently short and its tension is sufficiently large.

Supplementary References

1. Farhadifar, R., Röper, J.-C., Aigouy, B., Eaton, S. & Jülicher, F. The influence of cell mechanics, cell-cell interactions, and proliferation on epithelial packing. *Curr. Biol.* **17**, 2095–2104 (2007).
2. Spencer, M. A., Jabeen, Z. & Lubensky, D. K. Vertex stability and topological transitions in vertex models of foams and epithelia. *Eur. Phys. J. E Soft Matter* **40**, 2 (2017).
