# Supplementary material for: Multiciliated cells use filopodia to probe tissue mechanics during epithelial integration in vivo
Source: Nat Commun. 2022 Oct 28;13:6423. doi: 10.1038/s41467-022-34165-0 (PMC9616887; doi:10.1038/s41467-022-34165-0)
Supplement: Supplementary file 3 — Description of Additional Supplementary Files [file 41467_2022_34165_MOESM3_ESM.pdf]

## Description of Additional Supplementary Files

**File name: Supplementary Movie 1**

**Description: 3D rendering of integrating MCC interacting with epithelial vertices.**

MCC expressing α-tubulin::LifeAct-GFP (pseudo-colored in green) and goblet cells expressing nectin::Utrophin-RFP (pseudo-colored in magenta).

**File name: Supplementary Movie 2**

**Description: 3D plots for filopodia dynamics.**

Relative position of F-actin protrusions (magenta) extended by an integrating MCC (cyan) and the overlaying epithelial vertices (vertical tracks, color-coded for distance) during lateral movement.

**File name: Supplementary Movie 3**

**Description: 3D rendering of MCC interacting with epithelial vertices.**

Integrating MCC expressing α-tubulin::LifeAct-RFP (pseudo-colored in green) and goblet cells expressing LSR-3xGFP (pseudo-colored in magenta).

**File name: Supplementary Movie 4**

**Description: LSR localizes to filopodia tips.**

Integrating MCC expressing α-tubulin::LifeAct-RFP (pseudo-colored in green) and α-tubulin::LSR-GFP (pseudo-colored in magenta). Scale bar: 2 µm.

**File name: Supplementary Movie 5**

**Description: LSR depletion blocks MCC integration.**

Integrating control and LSR MO#1 MCCs expressing LifeAct-GFP (pseudo-colored in green). LSR-depleted cells using LSR MO#1 are marked with H2B-RFP (pseudo-colored in magenta). Scale bar: 20 µm.

**File name: Supplementary Movie 6**

**Description: LSR overexpression induces the formation of ectopic filopodia.**

Integrating MCC expressing α-tubulin::LifeAct-RFP (pseudo-colored in green) and α-tubulin::LSR-GFP (pseudo-colored in magenta). Scale bar: 5 µm.

**File name: Supplementary Movie 7**

**Description: 3D rendering of filopodia pulling on epithelial vertices.**

Integrating MCC expressing α-tubulin::LifeAct-RFP (pseudo-colored in green) and goblet cells expressing nectin::LSR-GFP (pseudo-colored in magenta).

**File name: Supplementary Movie 8**

**Description: Orthogonal view of filopodia pulling on epithelial vertices.**

Integrating MCC expressing α-tubulin::LifeAct-RFP (pseudo-colored in green) and goblet cells expressing nectin::LSR-GFP (pseudo-colored in magenta). Scale bar: 5 µm.

**File name: Supplementary Movie 9**

**Description: Rosette-like structure formation during MCC integration.**

Right, integrating MCCs expressing α-tubulin::LifeAct-RFP (pseudo-colored in green) and goblet cells expressing SF9-3xGFP (pseudo-colored in magenta). Left, segmented image with tracked cells pseudo-colored with different colors. Scale bar: 20 µm.

**File name: Supplementary Movie 10**

**Description: Laser ablation of epithelial goblet cell junction.**

Epithelial junctions are labeled with SF9-3xGFP (pseudo-colored in fire). Scale bar: 10 µm.

**File name: Supplementary Movie 11**

**Description: Junction remodeling during MCC integration.**

Integrating MCC expressing α-tubulin::LifeAct-RFP (pseudo-colored in green) and goblet cells expressing SF9-3xGFP (magenta). Scale bar: 5 µm.

**File name: Supplementary Movie 12**

**Description: Junction retraction after contact loss.**

Integrating MCC expressing α-tubulin::LifeAct-RFP (pseudo-colored in green) and goblet cells expressing SF9-3xGFP (pseudo-colored in magenta). Scale bar: 10 µm.

**File name: Supplementary Movie 13**

**Description: Orthogonal view of MCC vertex retraction after contact loss.**

Integrating MCC expressing α-tubulin::LifeAct-RFP (pseudo-colored in green) and goblet cells expressing LSR-3xGFP (pseudo-colored in magenta). Scale bar: 5 µm.

**File name: Supplementary Movie 14**

**Description: Myosin II is recruited to the leading edge of integrating MCCs.**

Goblet cells and MCC expressing SF9-3xGFP (pseudo-colored in magenta). Integrating MCC expresses MCC marker α-tubulin::LifeAct-RFP (pseudo-colored in green). Scale bar: 10 µm.

**File name: Supplementary Movie 15**

**Description: Myosin II downregulation blocks junction remodeling and cell integration.**

Goblet cells and CA-MYPT overexpressing MCC expressing LifeAct-GFP mRNA (pseudo-colored in green). CA-MYPT overexpressing MCC is labeled with H2B-RFP (pseudo-colored in magenta). Scale bar: 10 µm.
