# Supplementary material for: Multiciliated cells use filopodia to probe tissue mechanics during epithelial integration in vivo
Source: Nat Commun. 2022 Oct 28;13:6423. doi: 10.1038/s41467-022-34165-0 (PMC9616887; doi:10.1038/s41467-022-34165-0)
Supplement: Supplementary file 19 — Reporting Summary [file 41467_2022_34165_MOESM19_ESM.pdf]

nature research

Corresponding author(s): Jakub Sedzinski / Amin Doostmohammadi

Last updated by author(s): 01.10.2022

Reporting Summary

Nature Research wishes to improve the reproducibility of the work that we publish. This form provides structure for consistency and transparency in reporting. For further information on Nature Research policies, see our Editorial Policies and the Editorial Policy Checklist.

Statistics

For all statistical analyses, confirm that the following items are present in the figure legend, table legend, main text, or Methods section.

n/a	Confirmed	
☐	☒	The exact sample size (*n*) for each experimental group/condition, given as a discrete number and unit of measurement
☐	☒	A statement on whether measurements were taken from distinct samples or whether the same sample was measured repeatedly
☐	☒	The statistical test(s) used AND whether they are one- or two-sided<br>*Only common tests should be described solely by name; describe more complex techniques in the Methods section.*
☒	☐	A description of all covariates tested
☒	☐	A description of any assumptions or corrections, such as tests of normality and adjustment for multiple comparisons
☐	☒	A full description of the statistical parameters including central tendency (e.g. means) or other basic estimates (e.g. regression coefficient) AND variation (e.g. standard deviation) or associated estimates of uncertainty (e.g. confidence intervals)
☐	☒	For null hypothesis testing, the test statistic (e.g. *F*, *t*, *r*) with confidence intervals, effect sizes, degrees of freedom and *P* value noted<br>*Give P values as exact values whenever suitable.*
☒	☐	For Bayesian analysis, information on the choice of priors and Markov chain Monte Carlo settings
☒	☐	For hierarchical and complex designs, identification of the appropriate level for tests and full reporting of outcomes
☒	☐	Estimates of effect sizes (e.g. Cohen's *d*, Pearson's *r*), indicating how they were calculated

Our web collection on statistics for biologists contains articles on many of the points above.

Software and code

Policy information about availability of computer code

Data collection	Commercial Zeiss Zen Black v. 2.3 software was used to perform confocal imaging. Computer simulations were developed by the authors in Python version 3.7 and can be requested directly from the corresponding authors.
Data analysis	Data were analyzed with Fiji v. 1.53n, Matlab R2017b, and Python Pandas library using built-in functions. Graphs were generated using Matlab R2021b, Plot2 and Sigma Plot v.14/2018. The plugins used for segmentation and tracking (in Fiji) are also quoted in methods, including Tissue analyzer (https://github.com/baigouy/tissue_analyzer) and Cellpose (https://github.com/mouseland/cellpose). The Matlab pipeline used for analysis of filopodia is available on the following github link which is specified in the Code availability section of the manuscript (https://github.com/RaghavanThiagarajan/MCC_filopodia_quantification).

For manuscripts utilizing custom algorithms or software that are central to the research but not yet described in published literature, software must be made available to editors and reviewers. We strongly encourage code deposition in a community repository (e.g. GitHub). See the Nature Research guidelines for submitting code & software for further information.

Data

Policy information about availability of data

All manuscripts must include a data availability statement. This statement should provide the following information, where applicable:

- Accession codes, unique identifiers, or web links for publicly available datasets
- A list of figures that have associated raw data
- A description of any restrictions on data availability

The data and materials that support these findings are available within the article and its supplementary information files. Additional information and relevant raw data are available from the corresponding authors, Jakub Sedzinski (jakub.sedzinski@sund.ku.dk) and Amin Doostmohammadi (doostmohammadi@nbi.ku.dk) upon

request. Source data are provided with this paper.

Field-specific reporting

Please select the one below that is the best fit for your research. If you are not sure, read the appropriate sections before making your selection.

☒ Life sciences ☐ Behavioural & social sciences ☐ Ecological, evolutionary & environmental sciences

For a reference copy of the document with all sections, see nature.com/documents/nr-reporting-summary-flat.pdf

Life sciences study design

All studies must disclose on these points even when the disclosure is negative.

Sample size	No specific statistical technique was used to determine the sample size. The number of cells used for quantification was chosen based on the known variability of radial intercalation process and based on our previous experience (Sedzinski et al. Dev. Cell 2016, Sedzinski et al. JCS 2017). We observed higher cell to cell variability rather than an embryo to embryo variability in most of the radial intercalation process and as such average data from several embryos without distinction. The statistical variation of the data did not change considerably upon addition of more data points.
Data exclusions	No data was excluded
Replication	All experiments were replicated at least thrice and showed reproducible results. Independent experiments were performed and statistical analysis done independently of these data sets. The obtained results were the same within error bars in different, independent data sets.
Randomization	No specific experimental groups were defined and all data were considered.
Blinding	We have not sampled or analyzed data blindly as most of the experimental treatment was associated with clear phenotype. Data analysis was performed by automated software which was blind to data collection, which should remove any experimental bias. We have not used any specific sampling nor we had exclude any data.

Reporting for specific materials, systems and methods

We require information from authors about some types of materials, experimental systems and methods used in many studies. Here, indicate whether each material, system or method listed is relevant to your study. If you are not sure if a list item applies to your research, read the appropriate section before selecting a response.

Materials & experimental systems

n/a	Involved in the study	
☐	☒	Antibodies
☒	☐	Eukaryotic cell lines
☒	☐	Palaeontology and archaeology
☐	☒	Animals and other organisms
☒	☐	Human research participants
☒	☐	Clinical data
☒	☐	Dual use research of concern

Methods

n/a	Involved in the study	
☒	☐	ChIP-seq
☒	☐	Flow cytometry
☒	☐	MRI-based neuroimaging

Antibodies

Antibodies used	Anti-LSR (Angulin-1) Rabbit Polylclonal (1:50), Donkey Anti-rabbit Alexa647 (1:250) (ThermoFisher; A-31573), Donkey Anti-rabbit Cy3 (1:200) (Jackson Immunoresearch; 112-166-143), Anti-phospho Myosin Light Chain 2 (1:50) (Cell Signaling; 3671S)
Validation	The rabbit angulin-1/LSR antisera were raised against GST-angulin-1/LSR (a.a. 354-575) (Covance, Princeton, NJ). Antibody was affinity-purified using nitrocellulose membrane (Fisher Scientific, Pittsburgh, PA). Anti-LSR antibody was validated by independent publication (Higashi et al 2016). Negative control stainings were done as a control. Anti-phospho Myosin Light Chain 2 antibody has been used in previous publication in Xenopus (Reyes et al., Current Biology, 2014) and showed an obvious specificity to active myosin II. The rest of antibodies used in this study (secondary antibodies) are commercially available antibodies that have been extensively tested by manufacturing companies and also generally validated by researchers.

Animals and other organisms

Policy information about studies involving animals; ARRIVE guidelines recommended for reporting animal research

Laboratory animals	Xenopus laevis (5 years old) was used as an animal model. Since only embryos were studied, sex-specific experiments were not necessary, as Xenopus embryos at the studies stage have not yet undergone sex determination.
Wild animals	None
Field-collected samples	None
Ethics oversight	The National animal ethics committee in Denmark reviewed and approved all animal procedures (Permit number 2017-15-0201-01237).

Note that full information on the approval of the study protocol must also be provided in the manuscript.
